# Interactive 3D visualization of structural changes in the brain of a person with corticobasal syndrome

**DOI:** 10.3389/fninf.2014.00042

**Published:** 2014-05-05

**Authors:** Claudia Hänel, Peter Pieperhoff, Bernd Hentschel, Katrin Amunts, Torsten Kuhlen

**Affiliations:** ^1^JARA - High Performance Computing, IT Center - Computational Science and Engineering, Computer Science Department, Virtual Reality Group, RWTH Aachen UniversityAachen, Germany; ^2^JARA - Translational Brain Medicine, Institute of Neuroscience and Medicine (INM-1), Research Centre JülichJülich, Germany; ^3^C. and O. Vogt Institute for Brain Research, Heinrich Heine University DüsseldorfDüsseldorf, Germany

**Keywords:** volume rendering, view-dependent visualization, virtual reality, deformation-based morphometry, neurodegeneration, atrophy

## Abstract

The visualization of the progression of brain tissue loss in neurodegenerative diseases like corticobasal syndrome (CBS) can provide not only information about the localization and distribution of the volume loss, but also helps to understand the course and the causes of this neurodegenerative disorder. The visualization of such medical imaging data is often based on 2D sections, because they show both internal and external structures in one image. Spatial information, however, is lost. 3D visualization of imaging data is capable to solve this problem, but it faces the difficulty that more internally located structures may be occluded by structures near the surface. Here, we present an application with two designs for the 3D visualization of the human brain to address these challenges. In the first design, brain anatomy is displayed semi-transparently; it is supplemented by an anatomical section and cortical areas for spatial orientation, and the volumetric data of volume loss. The second design is guided by the principle of importance-driven volume rendering: A direct line-of-sight to the relevant structures in the deeper parts of the brain is provided by cutting out a frustum-like piece of brain tissue. The application was developed to run in both, standard desktop environments and in immersive virtual reality environments with stereoscopic viewing for improving the depth perception. We conclude, that the presented application facilitates the perception of the extent of brain degeneration with respect to its localization and affected regions.

## 1. Introduction

The simultaneous 3D visualization of both, the outer surface and internal structures of the human brain in an intuitively graspable manner is still challenging. The pattern of gyri and sulci of the outer brain surface provides landmarks for at least coarse localization. Besides, internal brain structures must be distinguishable due to their high functional specificity. Moreover, in neuroscience it is desired to combine such representations with different kinds of additional field data; thresholded maps of such field data shall be integrated with structural data. In the present study, the following types of field data were superimposed on a magnetic resonance imaging (MRI) scan of a brain of a patient who suffers from the corticobasal syndrome (CBS): Structural MRI data, time dependent field data that quantify structural changes on the voxel level, and probabilistic maps of anatomical regions (cf. Zilles et al., 2002; Amunts et al., 2007). The structural change data were calculated by analyzing series of longitudinally acquired MRI data using deformation based morphometry (DBM, cf. Pieperhoff et al., 2008). New insights into brain regions that are affected by certain neurodegenerative diseases are enabled by exploration of occurring structural changes and its temporal progression.

Visualizations of these brain data by means of 2D sections are widely used. However, each of these sections provides only a small cutout of the brain. Thus, it is left to the observer to mentally merge the information into a 3D representation. In particular, it is difficult to relate the information given in a 2D section to the cortical surface of an individual brain, e.g., to identify individual sulci. To this end, an additional 3D visualization can be provided separately or in combination with sections. Several software tools are available for the side-by-side 2D and 3D visualization of brain data. For example, Brainvoyager QX (http://www.brainvoyager.com/) is a commercial software specialized for functional MRI and diffusion tensor imaging (DTI), but the extension of this program for other data modalities is not straightforward because of special visualization needs. OpenWalnut (http://www.openwalnut.org/) and MITK (www.mitk.org) are open source toolkits that can be extended via plugins, or even within the code basis. Whereas OpenWalnut is specialized on DTI data, MITK is a tool for the processing of more general medical data. Both applications offer a 3D visualization complemented by 2D sections in different orientations.

The 3D visualization of the human brain, however, raises particular difficulties that are usually not considered by standard software. A transparent brain surface representation might become difficult to comprehend when the surface is overlapping too many times along the view direction. On the contrary, visualizing the brain as an opaque surface will occlude major parts of itself. For example, Thompson et al. (2007) and Zhou et al. (2013) use opaque surface renderings of the whole brain or of certain segmented structures that are colored by additional field data, such as volume change data or statistical scores. But any information inside or outside the rendered surfaces is discarded. To enable the representation of subcortical brain structures like the border between cortex and white matter, subcortical nuclei, or ventricles, 3D visualizations are often combined with up to three clipping planes (cf. Weber et al., 2008; Olabi et al., 2012). These planes define a clipping cuboid, in which the part of the brain inside this cuboid is removed and the clipping planes itself represent 2D sections. For the combined visualization of data from different modalities, the clipping can be limited to individual data sets as shown in Born et al. (2009) and Rieder et al. (2013), where only the anatomy inside the clipping cuboid is removed and fiber bundles stay visible. Additionally, a transparent representation of the brain in the cutout improves spatial perception of non-clipped structures.

Using a cuboid for the clipping, however, may require clipping too large parts of the brain, for example, when structures in the central part of the brain should be depicted. Therefore, a more flexible clipping geometry would be beneficial. As an alternative to the clipping planes, Rick et al. (2011) present a flashlight metaphor that enables the user to define interactively a cutout within the opaque volume visualization of the brain anatomy. Orientation, diameter, and depth of the clipping cone can be adjusted by the user. Still, this method is of limited use for elongated structures when observing the whole structure at once, because the diameter of the cone becomes unnecessarily large for the non-elongated direction and again too large parts of the brain might be clipped. Otherwise, a cone with a small diameter could be moved manually through the volume showing only a small part of the volume of interest (VOI) at once.

More data-driven visualization concepts are presented, e.g., by Hauser et al. (2001), Krüger et al. (2006), Bruckner et al. (2006), and Viola et al. (2004). These designs have in common that they locally decrease the opacity of occluding structures to show internal ones. Hauser et al. (2001) suggest to render different structures of a data set as individual objects separately in a first step. These objects are merged in a second step in order to give each of them a customized appearance in the final visualization. To focus on a small part of the whole volume, Krüger et al. (2006) use focus and context techniques and apply different weights, transparency functions, or color properties for focus and context objects. Bruckner et al. (2006) motivate their concept by hand-drawn illustration techniques and influence the focus mainly by defining the distance to the eye point and a gradient magnitude. The user can influence the sharpness of transition between clipped and visible structures, and the depth of clipping. The drawback of the three previously described techniques is the missing depth information for the VOI. Viola et al. (2004) resolve this problem with a technique using a conical cutout, that is comparable to the flashlight metaphor of Rick et al. (2011). The cutout shows anatomical information on its faces, thus giving depth information. This importance-driven volume rendering (IDVR) approach has the advantage that the cutout can be assigned to a particular structure, so that it can be automatically adjusted to the VOI's size. Furthermore, the cutout follows the view direction of the user and therefore stays always perfectly aligned. But for neuroscientific applications this technique might be further improved by providing additional information at the same depth as the VOI creating a section-like view onto the data and a data-depended clipping object for the deformation data.

**Figure 1:**
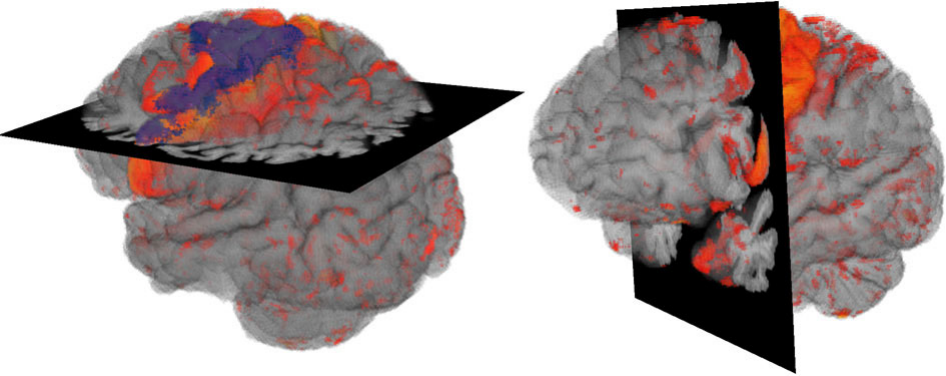
**Overview Design: Volume visualization showing brain degeneration (yellow/red) and the premotor cortex area (blue) in anatomical context (gray)**.

**Figure 2:**
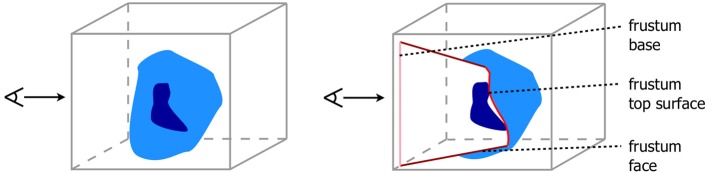
**View dependent frustum-like cutout into the volume (light blue) following the depth structure of the VOI (dark blue)**.

**Figure 3:**
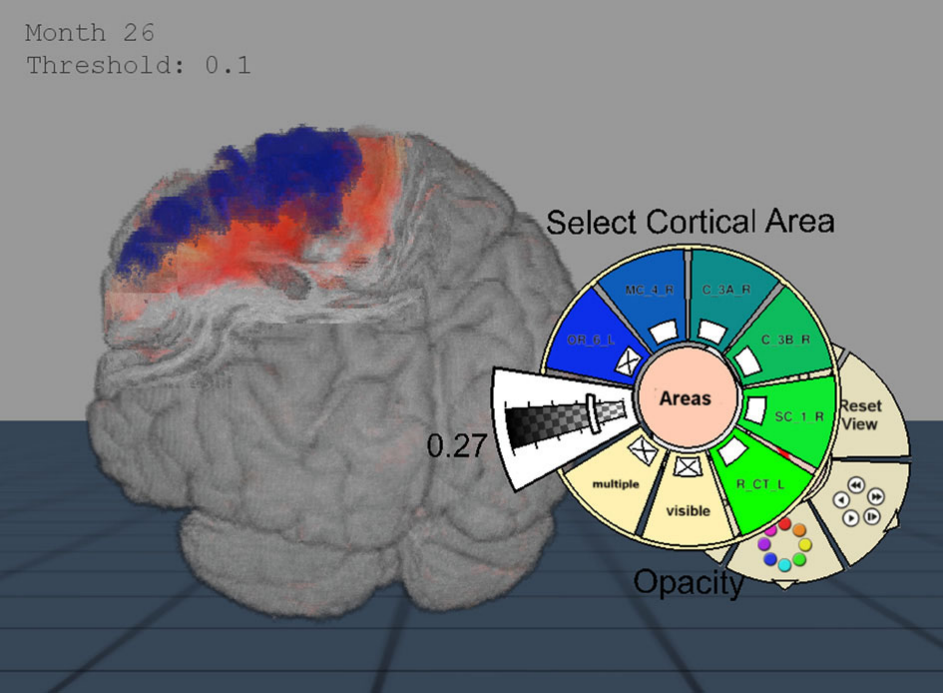
**Importance-Driven Volume Rendering Design: A view-dependent cutout is created to the premotor cortex area (blue)**. Furthermore, this screenshot shows the application when changing the opacity of the cortical area via pie menu.

Based on the previous findings we introduce two designs for the visualization of brain data with time dependant structural changes. The user interface of our visualization system had to enable an intuitive interaction and to provide an overview of the whole data in combination with detailed view of spatial relations of anatomical structures. The first design provides detailed anatomical information by means of a transparent anatomy of a whole MRI brain data set, whereby a 2D section can be interactively defined within this volume (cf. Figure 1). The second design extends the approach of Viola et al. (2004) by using a frustum of a cone as clipping object (cf. Figure 2) to provide more context information about nearby structures on the clipping planes (cf. Figure 3).

The rest of the paper is structured as follows: In section 2 image data as well as details of our visualization designs and interaction strategies are described. In section 3, the benefits of our implementation are discussed and in section 4 conclusions are drawn and an outlook onto possible future improvements is given.

## 2. Methods

Before we describe the two visualization designs mentioned above in detail, the underlying data modalities are clarified. Furthermore, we show how the user interface of our visualization system aims for an intuitive interaction and provide both, an overview of the whole data and a detailed view of spatial relations.

### 2.1. Data and image analysis

In this work a series of T1-weighted MR-images of a single person was used as an exemplar. The images were acquired by Südmeyer et al. (2012) in the context of a longitudinal study on aging and neurodegenerative diseases. The voxel-size of the MR-images was 1 × 1 × 1 mm^3^. These images were acquired at five points in time within a total interval of 26 months. The initial MR-image was segmented by deleting the value of every voxel not belonging to brain tissue. Segmentation masks were automatically generated by a procedure which was implemented in the program SPM (http://www.fil.ion.ucl.ac.uk/spm/) and afterwards manually corrected. Maps of volume changes, which were superimposed to the structural image, were calculated by DBM in the following way. Each follow-up MR-image of the subject was non-linearly registered with the initial image by minimizing the voxel-wise squared intensity differences between both images, regularized by an elastic energy term which penalized non-biological distortions. The image registration yielded for each follow-up MRT image a deformation field that assigned to each voxel of the initial MRT image a vector that pointed to the corresponding position in the follow-up image. From this deformation field, a map of voxel-wise relative volume differences was derived. Further details of this analysis can be found in Pieperhoff et al. (2008). In order to visualize the temporal evolution of tissue degeneration fluently, volume change maps in-between the actual time points–month 0, 16, 20, 23, and 26–were interpolated to a total number of 27 data sets.

Maps of anatomical regions used here originate from the JuBrain Cytoarchitectonic Atlas (https://www.jubrain.fz-juelich.de). They were gained by cytoarchitectonic based parcellations in histological sections of post-mortem brains (cf. Zilles et al., 2002; Amunts et al., 2007).

The anatomical data, time dependent field data and cortical areas were used to develop the visualization designs presented below and were a use case to examine the supportive effect in the visual analysis of these data.

### 2.2. Visualization

We developed two different visualization designs to support the spatial understanding of the data. The first design used a transparent 3D representation of the anatomy and an opaque section. The second design was based on the IDVR algorithm as described in Viola et al. (2004) creating a view-dependent cutout around a defined VOI. In both designs, the degeneration of the brain tissue was visualized by means of time varying data, which were mapped to a red to yellow color map, with red meaning small and yellow large volume decline (cf. Südmeyer et al., 2012). Additionally, in order to identify the affected brain structures, maps of selected anatomical regions of the JuBrain atlas were included. For the visualization design described below, we had have to follow the requirement to present internal structural information with respect to external anatomy in a meaningful way. The use of volume rendering and an interactive adjustment of opacity values for each data set facilitated, for example, a visualization of structural changes caused by tissue atrophy and anatomical regions. Furthermore, the combined ray casting for all data in one volume renderer enabled a correct depth perception. Based on this, our first visualization design combined common modalities and was used particularly as an overview visualization, whereas the second design allowed for a detailed examination of a selective VOI.

#### 2.2.1. Overview design

2D sections can be combined with 3D visualizations when using them as clipping planes (cf. Cabral et al., 1994; Rößler et al., 2006; Rick et al., 2011) to assist spatial orientation. Our proposed overview design was based on this idea: The brain anatomy was shown semi-transparently by the use of volume rendering and complemented by a 2D section of the original MRT data. Thus, both, the complex structure of the brain surface with gyri and sulci as well as internal regions remained visible, and no information in front of the section was lost as with clipping planes. To give an overview on the tissue degeneration, the deformation data were blended into the 3D anatomy volume. The final design can be seen in Figure 1.

#### 2.2.2. Importance-driven volume rendering design (IDVR design)

In comparison to the overview design, the IDVR design offered a more specialized view for a detailed examination of specific brain areas. The anatomy was visualized in an opaque fashion and a cutout facilitated a view into the volume by removing only as much anatomy as necessary and staying automatically aligned to the user's view direction. For this purpose, the design used an advanced algorithm based on Viola et al. (2004), so that the specified VOI was always visible due to a view-dependent cutout. The original work defines a conical cutout, with the tip of the cone being determined by the VOI's deepest voxel along the view direction. The faces of the cone help to determine the depth of the VOI in the overall volume. However, a drawback of this previous algorithm was, nearby structures at the same depth of the VOI may be covered by the surrounding brain tissue. Therefore, it was more favorable to expand the cutout by using a section-like plane that is aligned with the back side (in viewing direction) of the VOI. Thus, Viola's algorithm was modified by using a frustum shaped cutout instead of a conical one. The top plane of this frustum was positioned at the level of the deepest VOI voxel. In the present study, the VOI was defined by a neuroanatomical region. Neuroanatomical regions, e.g., cortical areas or nuclei, have often a complex structure, so that its depth texture is strongly varying. Hence, creating the section only on basis of one depth value would neglect nearby structures on all other depth levels of the VOI. Therefore, we adapted the algorithm to define the top surface of a frustum-like cutout with a section that is approximated using all values of the VOI's backface (cf. Figure 2).

The cutout calculation was based on multi-pass raycast rendering and worked as follows. In the first pass, a special modification of a depth texture of the VOI was defined as not only the depth values are interesting, but also the exact sample position in the volume. Therefore, rays were directed into the volume that were defined by a previously calculated ray entry points texture *T*_*R*_ and a ray exit point texture *T*_*E*_. Along each ray, the *x*-, *y*-, and *z*-coordinate of the deepest VOI voxel and the accumulated length *l*_*a*_ until this point were determined. These values were stored into the output texture *T*_*V*_ of this first rendering pass. If the ray did not hit the VOI at all, the four texture element (texel) values were set to zero.

**Figure 4:**
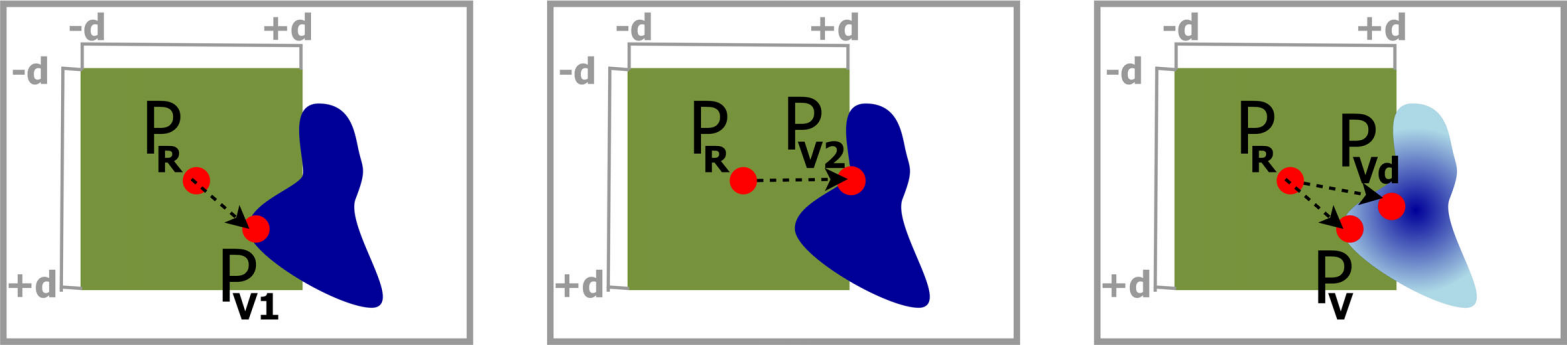
**Determination of the local depth value in the cutout. Left:** Use depth of *P*_*V*_1__ as the closest texel of the VOI's depth texture to the ray entry point *P*_*R*_. **Middle:** *P*_*V*_2__ is the most straight aligned texel in relation to *P*_*R*_. **Right:** A darker color in the VOI depicts a higher depth value. *P*_*V*_ is the closest texel to *P*_*R*_ and is used to calculate the distance to the VOI, but *P*_*V*_*d*__ has the highest depth value in distance *d* around *P*_*R*_, and is utilized as depth value.

In the second rendering pass, the cutout was defined. To find the best definition of the top surface of the frustum with respect to the best information retrieval and smoothness, three different implementations were tested. The first two approaches of the top surface definition varied only in the determination of the texel *P*_*V*_ ∈ *T*_*V*_ that is used as reference point for further calculations (cf. Figure 4 left, middle). In the first case a vector $\vec{P_{R}P_{V_{1}}}$ was sought, where *P*_*R*_ ∈ *T*_*R*_ was the current ray entry point and *P*_*V*_1__ ∈ *T*_*V*_ is defined by the closest texel of *T*_*V*_ with *l*_*a*_ > 0, within a maximum distance *d* in *X*- and *Y*-direction of *T*_*V*_. Therefore, the algorithm iterated over all texels of *T*_*V*_ from −*d* to +*d* distance in *X*- and *Y*-direction starting from the texel with the same texel coordinates as *P*_*R*_.

In the second case, we adapted this iteration step by not minimizing $\left|\vec{P_{R}P_{V_{1}}}\right|$, but rather find the texel *P*_*V*_2__ ∈ *T*_*V*_ that created a vector $\vec{P_{R}P_{V_{2}}}$ with a minimum angle between $\vec{P_{R}P_{V_{2}}}$ and the *X*- or *Y*-axis. The iteration starts at 0, checks in ±*d* in *X*- and *Y*-direction, and terminates if a sufficient *P*_*V*_2__ is found. From this point on, the calculations were identical for the first and second implementation and we defined *P*_*V*_ = *P*_*V*_1__ or *P*_*V*_ = *P*_*V*_2__, respectively.

Let *r*_1_ be the maximum length of $\vec{P_{R}P_{V}}$, with

(1) $$r_{1}=\sqrt{d^{2}+d^{2}},\text{ where }\sqrt{d^{2}+d^{2}}\geq |\vec{P_{R}P_{V}}|.$$

**Figure 5:**
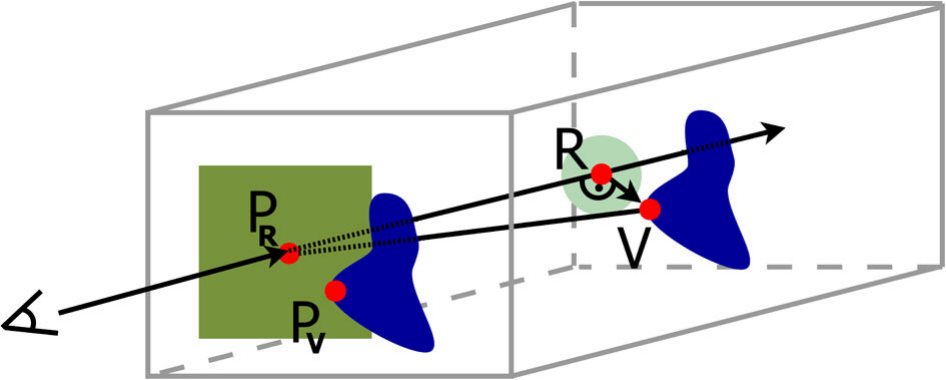
**Schematic illustration of the construction from the closest depth point of the volume of interest *V* onto the view ray *R***. In dark blue we see in the back the volume of interest and its projected depth texture on the near clipping plane. The dark green area limits our search area from the ray entry point *P*_*R*_ to a nearby VOI point *P*_*V*_, and the light green circle with radius *r*_2_ limits the top surface size.

If $\vec{P_{R}P_{V}}$ existed, it was possible to determine a vector $\vec{RV}$, with *V* being the corresponding voxel of the VOI to *P*_*V*_ saved in the output texture of the first rendering pass *T*_*V*_, and *R* being defined with the help of the congruence theorem of triangles, where an edge with an angle of 90 ° could be constructed from the view ray to *V* (cf. Figure 5). If $\left|\vec{RV}\right|$ was within a radius *r*_2_, with *r*_2_ ≤ *r*_1_, the ray hit the top surface of the frustum and the depth value *c* of the cutout for the current view ray was set to the depth value of *V*. Otherwise, the cutout depth *c* was calculated as follows

(2) $$c=|\vec{RV}|-\frac{|\vec{P_{R}P_{V}}|-r_{2}}{r_{1}-r_{2}}.$$

**Figure 6:**
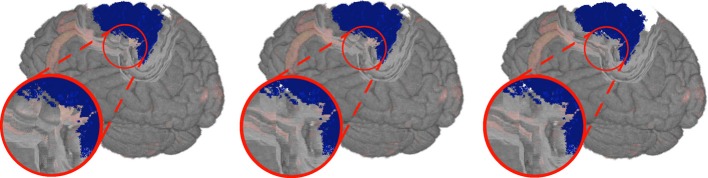
**Clipping artifacts depend on the definition of the distance value and are clearly visible when observing transitions in the sulci (dark gray) in the detail view. Left:** Circular artifacts when using the closest voxel of the VOI. **Middle:** Diagonal artifacts when preferring voxels in straight alignment. **Right:** Smoothest result with homogeneous depth values for a nearby voxel.

The result of the first approach showed circular artifacts around small parts of the VOI that stuck out, and where depth changes of the VOI occurred (cf. Figure 6 left). For the second approach, we see hard edges in diagonal orientation (cf. Figure 6 middle). To create a smoother frustum top surface, neglecting small outliers, we implemented a third approach which is schematically shown in Figure 4 right. In addition to *P*_*V*_1__ the texel *P*_*V*_*d*__ in *TV* was sought within a maximum distance ±*d* to *P*_*R*_ in *X*- and *Y*- direction with the highest *l*_*a*_ value. $\vec{P_{R}P_{V}}$ was calculated as in the previous approaches, but $\vec{RV}$ was replaced with $\vec{RV_{d}}$ and the additional depth had to be included in the calculation of the frustum faces, resulting in

(3) $$c=|\vec{RV_{d}}|-|\vec{RV_{d}}|\cdot \frac{\left(|\vec{P_{R}P_{V}}|-r_{2}\right)}{r_{1}-r_{2}}.$$

Although the depth value of the top surface is determined by the depth value of *Vd*, $\left|\vec{RV}\right|$ still defines whether the view ray hits the top surface or is part of a frustum face. An exemplary smoothed cutout can be seen in Figure 6 right.

### 2.3. Interaction

Depth perception can be improved by rotating, panning, and zooming (cf. Swanston and Gogel, 1986), suggesting that interaction with the brain model in the 3D visualization is desirable. In immersive virtual environments, correct depth relations can already be perceived without additional intentional interaction. Therefore, we provided the application for both, standard desktop setups and 3D immersive virtual environments with stereoscopic vision. To this end, we used the open source, cross-platform ViSTA toolkit (cf. Assenmacher and Kuhlen, 2008) for easy scalability to different systems. In the immersive setup, the depth impression of the cutout in the IDVR design became better comprehensible and the location of the border between faces and top surface of the frustum was clearly visible. The advantage of virtual environments over 2D displays for depth perception and estimation were shown in several studies, e.g., in Armbrüster et al. (2006) and Naceri et al. (2010) and in particular for volume rendered data in Laha et al. (2012).

Because the application was provided for Virtual Reality setups, an alternative to the classical 2D menu interaction became necessary. To this end, we decided to use extended pie menus as described by Gebhardt et al. (2013). They scale to 2D and 3D environments and can interactively be moved in the scene while staying aligned to the user's orientation. The menu is hierarchically arranged and can be divided into various submenus (cf. Figure 3). The most important interactions that were controlled via these menus are explained below.

*Time Navigation*–This submenu held the time navigation, where the user was able to set the animation speed or can manually step through all time steps of the presented data.

*Cortical Areas*–Predefined anatomical regions such as cytoarchitectonic areas from the JuBrain atlas could be selected for visualization by this submenu. Color codes of these regions and their arrangement in groups could be defined in a separate settings file that was read when the program starts. These definitions were also represented in the pie menu, allowing the user to hide or display whole area groups with a single mouse click. Furthermore, the opacity could be adjusted to provide a view onto degeneration occurring inside the anatomical regions.

*Importance Driven Volume Rendering*–Here the user could switch to the IDVR design. Depending on the available data sets of the subject, a variety of options existed. By default the VOI was defined by the visible anatomical regions. If provided the user was able to select any other VOI defined by field data as well. It can be useful to show other data next to the VOI in the cutout. Therefore, the user might choose to visualize the degeneration, or if provided, any other data exempt from the anatomy.

*Color Map*–This submenu allowed users to adapt the opacity and enhance the contrast of the anatomy. The contrast and opacity parameters have to be adapted to the range of voxel values of the data sets which are to be visualized. Furthermore, the color menu allowed users to change the threshold for the deformation values to exclude small degeneration values and set the focus on larger ones. The adjustability of the opacity for these values led to a good spatial orientation particularly in the IDVR design because the degeneration could be visualized in the cutout and a high transparency preserves a good view onto the cutout faces. Moreover, in the overview design the right balance between opacity of cortical areas and degeneration allowed for good visual comparability of relationship between the two volumes.

## 3. Results and discussion

The visualization of anatomical structure and superimposed field data by volume rendering enables neuroscientists to observe the data described in section 2.1 not only on the surface, but also inside the brain, and to get a better impression about the spatial extent of regional volume loss in the context of the individual brain anatomy. This is an advantage over visualization based on surface reconstruction, because the latter is typically limited to field data on or near the surface, which causes a great loss of information. In studies of neurodegenerative diseases, it makes an important difference if the tissue atrophy that is quantified by the superimposed volume change data occurs only in the cortex, or if also subcortical regions and white matter (i.e., fibers deep inside the brain which connect brain regions) are affected. For instance, the data examined here show a progressive atrophy, which includes both the motor cortex areas and the pyramidal tract. But transparent volume rendering of the brain often yields diffuse borders, whereas in surface based visualization the perception of the surface shape can be enhanced, for example, by the simulated effects of lighting, reflection, and shadows. Moreover, brain structures that are deep inside the brain and have only low contrast to their environment are hardly perceived when volume rendering of an MRT image is used exclusively.

A 2D section is added in the overview design onto which the voxel values of the structural image of the brain are mapped, so that cortex, subcortical nuclei, and white matter can easily be identified according to existing brain atlases. The brain in front of the added plane is not removed and thus landmarks for an anatomical localization are still provided. This design is particularly useful, when certain features near or within the brain cortex like the atrophy of certain gyri shall be shown.

**Figure 7:**
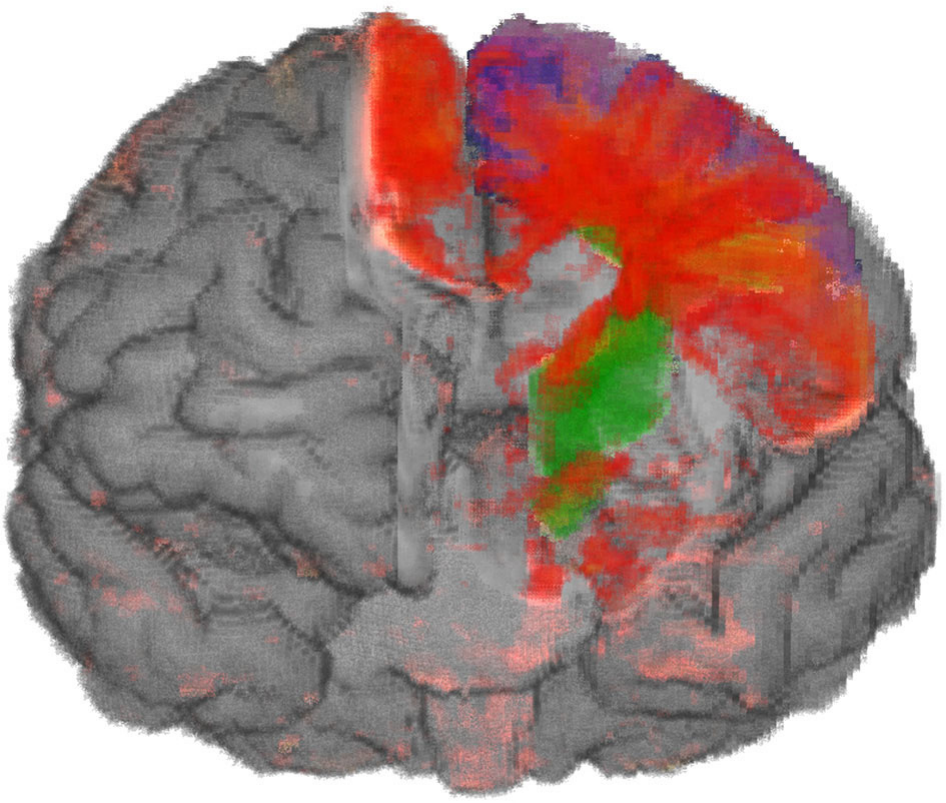
**Atrophic part of the brain (red to yellow) of a person with CBS and maps of anatomical regions (blue premotor cortex, green cortico-spinal tract)**. The removed part of the brain is adjusted to the selected anatomical regions. The overlap between atrophic parts and anatomical regions can be recognized by the blending of different colors.

In the IDVR design the brain in front of the clipping surface is removed, whereas the anatomical maps and the volume change data are still completely rendered. This design is more appropriate to show deeper parts of the brain: By means of rendering the anatomical regions in front of the clipping surface and the surface texture, a good localization is possible. In particular, the overlap of anatomical regions and volume change data is intuitively displayed by the blending of their colors (cf. Figure 7).

Moreover, the automatic alignment of the clipping surface when the brain is moved relative to the observer enables a simple interaction, which gives a good spatial perception even in non-virtual environments. The extent of the removed part of the brain can be controlled by the selection of predefined anatomical regions and additional parameters like the aperture of the frustum. We observed that using the application in a user-friendly immersive virtual environment enhances the perception of the spatial relations, in particular of spatial depth.

Since this application is to be used interactively, the frame rate has an important influence on the performance, which is why it was investigated in more detail. In comparison to visualizations of geometries, volume rendering approaches lack in performance. Moreover, the use of stereo viewing in an immersive virtual environment halves the frame rate due to the generation of two simultaneous images (one per eye). We tested our application on two systems: The first is used as desktop environment, and runs Windows 7 on Intel Xeon CPU E5540 with four cores at 2.5 GHz, an Nvidia GeForce GTX 480 graphics card, and 12 GB RAM; the second system is used as virtual environment with a passive stereo system, head tracking, and runs Windows 7 on Intel Xeon CPU E5530 with four cores at 2.4 GHz, but with an Nvidia Quadro 6000 graphics card, and 4 GB of RAM. With a resolution of 1400 × 1050 we achieve for the overview design about 30 frames per second (fps) in stereo mode which fulfills the requirement for interactivity. The implementation of the IDVR design is based on the same volume renderer, thus a higher frame rate cannot be expected. We are limited by the iteration over all neighboring texels when seeking *P*_*V*_ which it cannot be early terminated, because there is always the possibility to find a closer *V*, respectively, a *V*_*d*_ with higher *l*_*a*_. To solve this problem, we introduced a parameter to the IDVR calculation to set the accuracy in the iteration. Assuming that the resolution of the texture *T*_*V*_ is sufficiently large, every *i*-th texel can be skipped and is not tested to be a candidate for *V* or *V*_*d*_. The value for *i* can be changed via the pie menu. For *i* = 2 nearly no visual artifacts can be found. For *i* = 3 noticeable impacts in form of loose wrong depth values appear, but this method is still reasonable, because it allows for better performance and the artifacts are not disturbing the overall depth perception in the cutout. The achieved frame rates (cf. Table 1) for a scene comparable to the one shown in Figure 3 show that also the IDVR design can be used interactively in virtual environments, but clearly needs performance improvements. However, in general the frame rate of our application is highly dependent on the window size, the size of the volume on the screen, and for the IDVR design on the value of distance *d*.

**Table 1:** **Results of performance tests of the visualization designs (averaged values from different view points), the overview design with common volume rendering and the IDVR design with different precision states for the cutout generation**.

	**Nvidia GeForce GTX 480 (mono view)**	**Nvidia Quadro 6000 (stereo view)**
Overview Design	55 fps	30 fps
IDVR Design		
*i* = 1	4 fps	3 fps
*i* = 2	11 fps	7 fps
*i* = 3	18 fps	10 fps

## 4. Conclusion and future work

In this paper, we have introduced two visualization designs to address the challenge of depicting complex 3D information of human brain data. Whereas the first approach provides an overview of the data, the second allows for a more detailed examination. Current work as, for example, Laha et al. (2012) already showed that the visualization in virtual environments supports the analysis of volume data, but more studies are necessary in this field. First, the general improvement of spatial impression and the ability of correct spatial localization with our designs in comparison to commonly used 2D section views should be proven, for example, by determining the extension of an artificial tissue degeneration and its spatial localization. Second, this experiment should be repeated in an immersive virtual environment and then compared to the results of desktop environments. Moreover, since our application is supposed to benefit the daily workflow of neuroscientists, their expert impression of additional or more easily grasped information should be gathered.

Illustrations in anatomical text books like Nieuwenhuys et al. (2008) are excellent artworks that selectively emphasize certain structural entities or parts of the brain while showing the surrounding brain structure. These figures were artistic drawings, but it is desirable to achieve similar presentations by computerized 3D visualizations which can be manipulated by user interaction (cf. Bruckner et al., 2006). In particular, the gradient based emphasis of surface structures could be used to stress the brain surface and show the ventricles in the overview design more clearly. Furthermore, an enhanced contrast-to-noise ratio of the MRI data and a visual smoothing would improve the quality of the visualization and allow for easier analysis. As discussed in section 3, the IDVR design could benefit from a faster cutout calculation to ensure a higher frame rate which in turn would lead to increased interactivity. Therefore, one approach might be to use distance maps created from the result of the first rendering pass and discard the iteration approach during the second rendering pass that is mainly responsible for the frame rate decrease.

In conclusion, we have significantly improved the spatial localization of brain structures affected by CBS and the understanding of its temporal progression which motivates further research, and an application to other neurological and psychiatric disorders.

### Conflict of interest statement

The authors declare that the research was conducted in the absence of any commercial or financial relationships that could be construed as a potential conflict of interest.
